# Techniques for thoracic duct cannulation without thoracotomy in piglets

**DOI:** 10.1186/s12917-016-0808-3

**Published:** 2016-09-15

**Authors:** Hung-Hsun Yen, Helen M. S. Davies

**Affiliations:** Faculty of Veterinary and Agricultural Sciences, The University of Melbourne, Parkville, Victoria 3010 Australia

**Keywords:** Lymphatic cannulation, Thoracic duct, Pulmonary lymph, Swine

## Abstract

**Background:**

Pigs are the natural hosts of many zoonotic pathogens such as influenza viruses and *Staphylococcus aureus* and thus have advantages over non-natural hosts when studying these zoonotic diseases. In addition, pulmonary infections are a key issue in the pig industry, for example: porcine reproductive and respiratory syndrome virus infection. Exploration of the pathogenesis of swine pulmonary infections, in particular at the onset of disease, will provide valuable information for the development of vaccines against these diseases. Therefore, there is need to develop a methodology that allows for in vivo sampling of efferent pulmonary lymph with minimum damage to the target tissues for studying the pathogenesis of swine pulmonary infections.

**Results:**

We introduce the surgical procedures for cannulating the thoracic duct at its point of entry into the external jugular vein cranial to the first rib on the left in pigs. Using this methodology, we monitored the amounts of triglyceride and cholesterol in the lymph collected from the thoracic duct following 30 h fasting and at multiple time points after meals. It was found that the levels of triglyceride rather than cholesterol corresponded to the milky appearance of the lymph samples.

**Conclusions:**

Our techniques provide a strategy for collecting lymph including pulmonary lymph from the thoracic duct without thoracotomy. A pig model for collecting in vivo, in situ efferent lymph draining the lower respiratory tract and its local lymph nodes in real-time with minimal tissue damage to the target tissues opens a new door for studying disease processes in pulmonary infections. Techniques described here are the key to this door.

**Electronic supplementary material:**

The online version of this article (doi:10.1186/s12917-016-0808-3) contains supplementary material, which is available to authorized users.

## Background

Obtaining pulmonary lymph draining the lungs and the local lymph nodes (i.e. tracheobronchial lymph nodes) from the natural hosts of lung pathogens provides a strategy for monitoring in vivo*,* in situ pulmonary immunobiology and screening the biomarkers following pulmonary infections. Pigs are the natural hosts of several zoonotic pathogens for example influenza viruses (H1N1, H3N2, and H1N2) [1], Japanese encephalitis virus [2, 3] and *Staphylococcus aureus* [4], and thus are suitable for studying disease processes in these infections. Indeed, interest in using pigs as a model for influenza virus infection has increased since the outbreak of the swine-origin influenza A (H1N1) 2009 virus infection in humans [5, 6].

Pulmonary infections are a key issue in humans and in the pig industry [7]. For example, the economic loss to the pork producers caused by porcine reproductive and respiratory syndrome virus (PRRSV) infection was estimated to be US $664 million per annum [8]. To explore the pathogenesis of swine pulmonary infections, in particular at the onset of these diseases, will provide valuable information for the development of vaccines against these diseases. Real-time in vivo pulmonary lymph collection provides the chance to monitor the progress of an infection from the first contact of a pathogen with the host to the induction of illness. To achieve effective monitoring of pulmonary responses, a strategy for accessing in vivo pulmonary lymph samples in pigs is needed.

The structure of porcine lymph nodes is inverted compared to other species. Pabst and Binns also reported that most porcine lymphocytes leave lymph nodes via high endothelial venules (HEVs) instead of the efferent lymph [9]. However, the report by Binns in 1980 [10], indicates that, “In spite of the low cell numbers, efferent lymph responds to antigenic stimulation of the lymph node.”. Binns also mentioned that, ‘…efferent lymph flow and contents are normal in other respects…’. According to these previous findings, the efferent lymph supernatant of pigs will provide a useful source of samples for studying the components and immunity of the local lymph node and its draining tissues even though the numbers of white cells are relatively low. However, studies on the changes to the cells in porcine efferent lymph to infections at the draining tissues may be limited since it is unclear what type of lymphocyte egresses from lymph nodes via efferent lymph.

Techniques for cannulating the thoracic duct following thoracotomy by approaching its segment within the thoracic cavity have been described in pigs [11, 12]. However, surgical incisions into the chest wall result in severe tissue damage that can also induce immune responses other than those triggered by the pathogen in a pulmonary infection study. Due to this consideration, a model for harvesting pulmonary lymph without inducing severe tissue damage to the chest wall would be beneficial for respiratory studies.

From the anatomical layout, pulmonary afferent lymph from the lungs, in particular the caudal lobes of the lungs, drains into the tracheobronchial lymph centres and then enters the thoracic duct directly or through the cranial mediastinal lymph centre [13]. Anatomical dissection demonstrated that the thoracic duct extends cranially beyond the first rib, prior to terminating in the external jugular vein on the left in 12 of a group of 15 Large White piglets [14]. Therefore, procedures for catheterizing the thoracic duct can be developed by approaching the region where the thoracic duct joins the external jugular vein at the thoracic inlet.

Although techniques for thoracic duct cannulation without thoracotomy using different approaches have been developed in sheep [15, 16], the surgical approaches in pigs need to be re-developed due to noticeable anatomical variations [17]. For example, pigs have a prominent subclavian muscle lateral to the ampulla of the thoracic duct on the left. The porcine subclavian muscle arises from the second to fourth costal cartilage, courses dorso-laterally passing over the shoulder joint and joins with the aponeurosis of the supraspinatus muscle whereas the subclavian muscle of sheep is a narrow band, which joins the tendon of insertion of the brachiocephalic muscle. In addition, pigs have dorsal, middle and ventral superficial cervical lymph nodes in the caudal cervical region whereas only the superficial cervical lymph nodes are present in sheep. The locations of these lymph nodes also vary in these two species. Considering the anatomical variations between sheep and pigs, surgical procedures for thoracic duct annulation in sheep cannot be directly applied to pigs. Surgical procedures to achieve an approach to the thoracic duct with minimum damage to local tissues in pigs need to be studied and tailored based on porcine anatomy.

Here, we describe the surgical procedures developed for cannulating the thoracic duct with minimum tissue damage and without thoracotomy in pigs.

## Methods

### Experimental animals

The University of Melbourne Animal Experimentation Ethics Committee approved all experimental procedures. The methods were carried out in accordance with the approved guidelines.

Four female Large White piglets (aged 4–9 weeks, live weight range 9.3 to 17.1 kg) were housed in pens within the Faculty of Veterinary and Agricultural Sciences animal facility. These pigs were fed with commercial pellets (1 kg/day) and allowed access to water ad libitum. Following surgery, one pig was housed in a metabolic cage to reduce the possibility of wound damage and cannula removal. The other pigs were housed in pens post-surgery. At the end of the study, they were euthanized by intravenous injection of Lethabarb (Pentobarbitone) into the external jugular vein.

One pig was euthanized to obtain the anatomical images of the thoracic duct and the left tracheal trunk at their points of entry into the external jugular vein at the cervico-thoracic junction. Three pigs of different ages were used to develop the surgical procedures for thoracic duct cannulation without thoracotomy.

### General surgical procedures

#### Anaesthetic protocol

Prior to surgery, pigs were fasted overnight and provided water ad libitum. Anaesthesia was induced by intramuscular injection of Atropine (0.054 mg/kg) + Ketamine (15 mg/kg) + Xylazil (1 mg/kg) and then maintained with isoflurane (1.5–2.5 %) and oxygen using a mask. To prevent hypothermia, the pigs were placed on a heating pad in surgery. Each pig received an intramuscular injection of procaine penicillin (1 mL dose) and analgesic (Temgesic, 0.1 mg/kg) while under anaesthesia. Another dose of analgesic was given at day one post-surgery.

#### Cannulas

We used cannulas, SV28 (internal diameter 0.40 mm; external diameter 0.80 mm; CBAS-coated, Carmeda AB, Stock-holm, Sweden) and SV45 (internal diameter 0.58 mm; external diameter 0.96 mm) of different bores for pigs of different body weights [16]. The SV28 cannula was only applied to the cannulation surgery in a 5-week-old pig that weighed 9.3 kg. For an 8-week old pig, which weighed 17.1 kg and a 7-week old pig, which weighed 14.7 kg, we used the SV45 cannulas for the surgeries.

### Triglyceride and cholesterol measurement

Quantitation of triglycerides and cholesterol was carried out by Gribbles Veterinary Pathology (Melbourne, Australia).

## Results

**Fig. 1:**
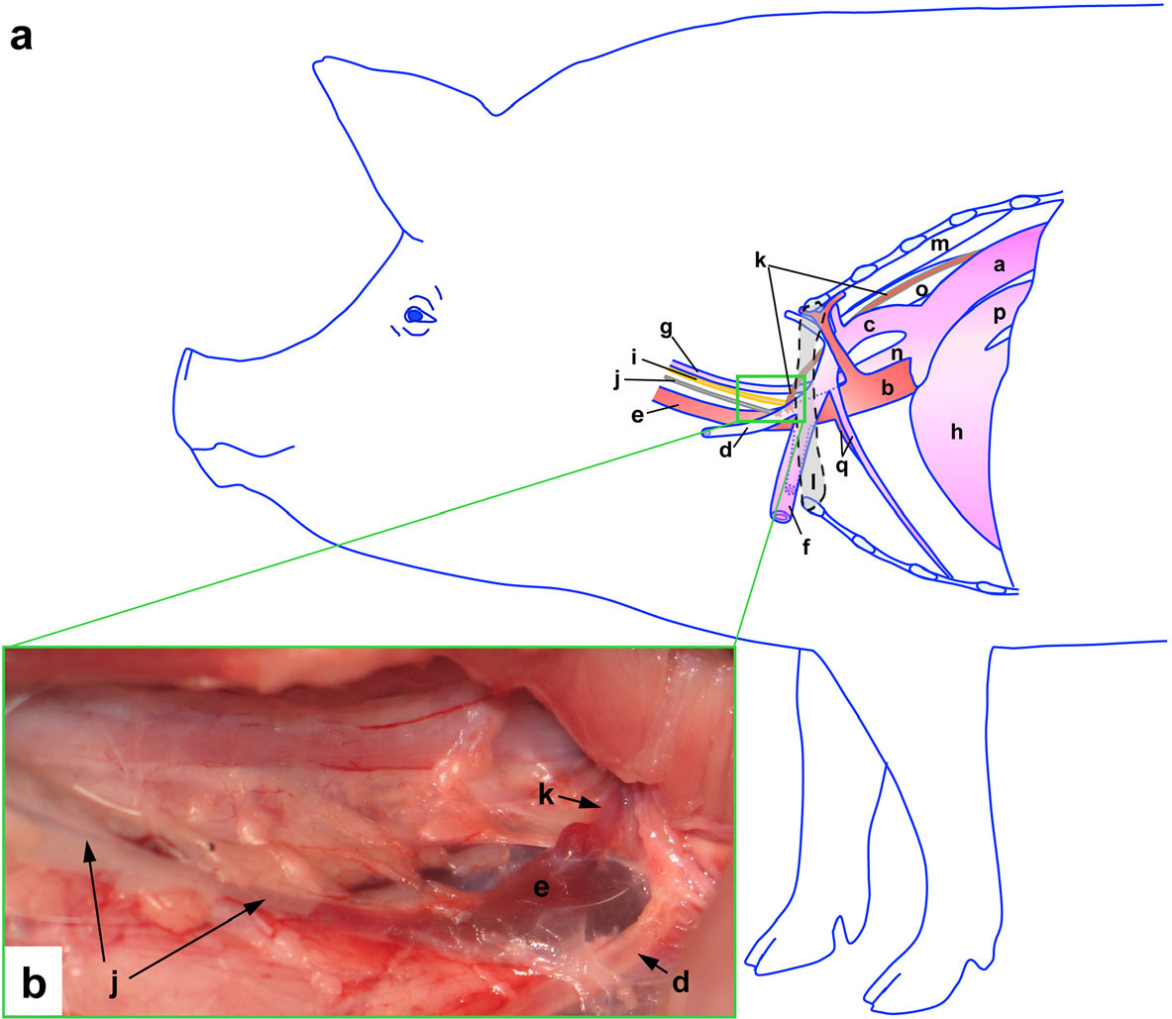
Schematic view (**a**) and photograph (**b**) of the thoracic duct and the tracheal trunk in the cervicothoracic region on the left side. (**a**) The thoracic duct containing lymph draining the lungs emerging from the thoracic cavity joins the external jugular vein at the cranial thoracic inlet. The ampulla of the thoracic duct is located craniomedially to the first rib (l). The thoracic duct (k) is medial to the subclavian and superficial cervical arteries (c & d respectively) and lateral to the common carotid artery (g) and vagosympathetic trunk (i). (a) thoracic aorta; (b) cranial vena cava; (c) subclavian artery; (d) superficial cervical artery; (e) external jugular vein; (f) axillary artery; (g) common carotid artery; (h) heart; (i) vagosympathetic trunk; (j) tracheal trunk; (k) thoracic duct; (l) first rib; (m) longus colli; (n) brachiocephalic trunk; (o) oesophagus; (p) pulmonary trunk. (**b**) Dissections were performed from the cranial border of the left forelimb to expose the cranial thoracic inlet. The superficial cervical artery (d) was retracted to reveal the thoracic duct (k) and its point of entry into the left external jugular vein (e). The tracheal trunk (j) coursing caudally from the medial retropharyngeal lymph centre also entered the left external jugular vein (e). The image was taken from a euthanized 7-week old Large White pig

Other than the information provided in Fig. 1, the detailed anatomical structures in the left cervicothoracic region and the left forelimb in pigs were acquired from textbooks [13, 18, 19]. The basic technique for inserting a cannula into a lymphatic has been described in previous publications [16, 20, 21]. As a result, unless the procedures were essential for this surgery, they are not repeated here.

The animal was positioned in right lateral recumbency. After a surgical incision in the skin cranial to the prescapular region, dissections were conducted towards the thoracic inlet to approach and isolate the thoracic duct. The skin incision of about 13 cm in these pigs was commenced from the cranial boundary of the subclavian muscle and its junction with the omotransversarius muscle and extended ventrally to about 2 cm cranial to the shoulder joint. After cutting through the superficial fascia, dissection was required to penetrate through the subcutaneous tissue and fat to separate the subclavian muscle from the cleidooccipitalis part of brachiooccipitalis muscle. This was mostly performed through blunt dissection to avoid damaging the main blood vessels, nerves, lymphatics and muscles.

Branching from the superficial cervical artery and vein, there were generally two blood vessels that coursed caudally in the subcutaneous tissue and fat to the supraspinatus region. Consequently, care was required to isolate these large blood vessels and ligate them. Transecting these blood vessels provided more space to approach the deeper tissues. After transecting these blood vessels, two self-retained retractors were placed in the skin opening to generate an operating area and to improve access to the thoracic duct.

Blunt dissection was then used to penetrate the connective tissues and locate the superficial cervical artery and vein, the dorsal superficial cervical lymph nodes (SCLD) and the efferent lymphatic of the SCLD. The efferent lymphatic of the SCLD drains into the external jugular vein and the point of its entry to the external jugular vein was next to the ampulla of the thoracic duct. Consequently, one approach to find the junction of the thoracic duct and the external jugular vein was to follow the efferent lymphatic of the SCLD as well as the superficial cervical artery that runs parallel to it. The other key lymphatic that needed to be identified in this region was the tracheal trunk. The tracheal trunk lies deeper in the fat and the connective tissues between the forelimb and the trachea. From our experience in pig dissections, the tracheal trunk was obviously bigger than the efferent lymphatic of the SCLD. Pigs might sometimes have double tracheal trunks [22]. When approaching and isolating the thoracic duct and its ampulla, we avoided damaging the tracheal trunk and the efferent lymphatic of the SCLD. Leaking lymph in the surgical area would have increased the difficulties in structure identification and cannula insertion.

The brachial plexus had nerve branches crossing the axilla from the neck musculature to the forelimb on the left. Great care was needed to avoid cutting the nerve branches of the brachial plexus when doing blunt dissections to isolate the thoracic duct and its ampulla. In particular, muscular nerve branches from the cervical nerve, C6/C7 to the subscapular region crossed the region of dissection. We opted to make space to approach the thoracic duct from the connective tissues and fat dorsal to the first big nerve branch that crossed to the subscapular region (usually a branch of C6/C7 that innervated the subscapular muscles). At day one post-surgery, all pigs were able to actively move around including climbing on the fences of the metabolic cage using the leg on the surgical (left) side. This outcome supported the conclusion that the surgical procedures we opted for did not result in damage to these nerve branches.

**Fig. 2:**
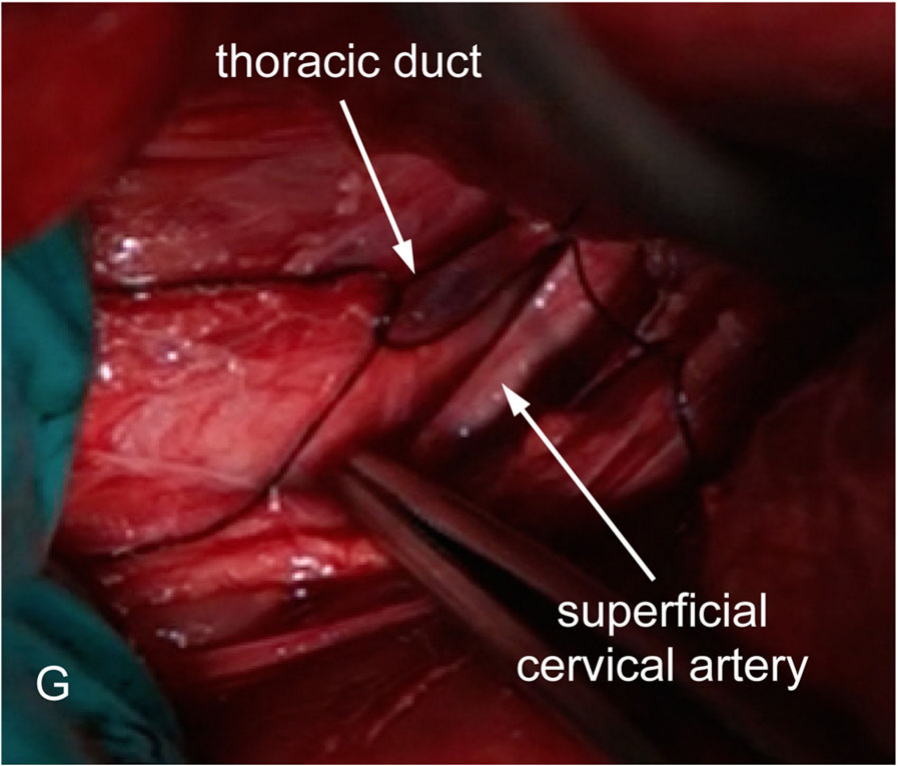
Intraoperative photograph of the surgical field. Surgery was conducted to separate a segment of the thoracic duct and its ampulla at the cranial thoracic inlet. The thoracic duct was gently isolated and two pre sutures were placed around it (Only one pre suture that is around the ampulla is shown in this image). The superficial cervical artery was retracted ventrally using a pair of forceps to reveal the thoracic duct. The size of the ampulla changed depending on the amount of blood in it. The ampulla is in a collapsed state in this image (G: gloves)

When the thoracic duct and its ampulla were identified, a segment of the lymphatic vessel was isolated that was enough to place two overhead knots around it. Isolation of this segment of the lymphatic was a critical step and there were some structures near the thoracic duct that ought to be noticed and should not be damaged. Figure 1 illustrates a schematic view (a) and a photograph (b) of the thoracic duct and its relationships to the adjacent structures in the cervicothoracic region on the left. As indicated in Fig. 1a, the left vagosympathetic trunk and common carotid artery were medial to the thoracic duct and they ran together with the thoracic duct through the thoracic inlet into the thorax. In addition, the superficial cervical artery was directly on the top of the thoracic duct and the ventral portion of the thoracic duct contacted the wall of the external jugular vein (Fig. 1a & Fig. 2). Figure 1b presents the thoracic duct and the tracheal trunk at the cranial thoracic inlet with the superficial cervical artery retracted to expose their points of entry into the external jugular vein on the left side of a euthanized animal. In addition, the placement of the suture around the thoracic duct had to be conducted with care. We used long forceps with blunt ends to separate the thoracic duct from the external jugular vein underneath and to place the suture. At least, two pre sutures were required to be placed around the thoracic duct. One was to ligate the thoracic duct to prevent bleeding from the external jugular vein. The other was to secure the cannula after its insertion into the thoracic duct. If there was enough space to do so it would be helpful to add an additional suture to secure the cannula. An intra-operative image of the surgical field with pre sutures around the thoracic duct is presented in Fig. 2. In this image, the superficial cervical artery was retracted ventrally using a pair of forceps to reveal a fragment of the thoracic duct and its ampulla for cannula insertion.

**Fig. 3:**
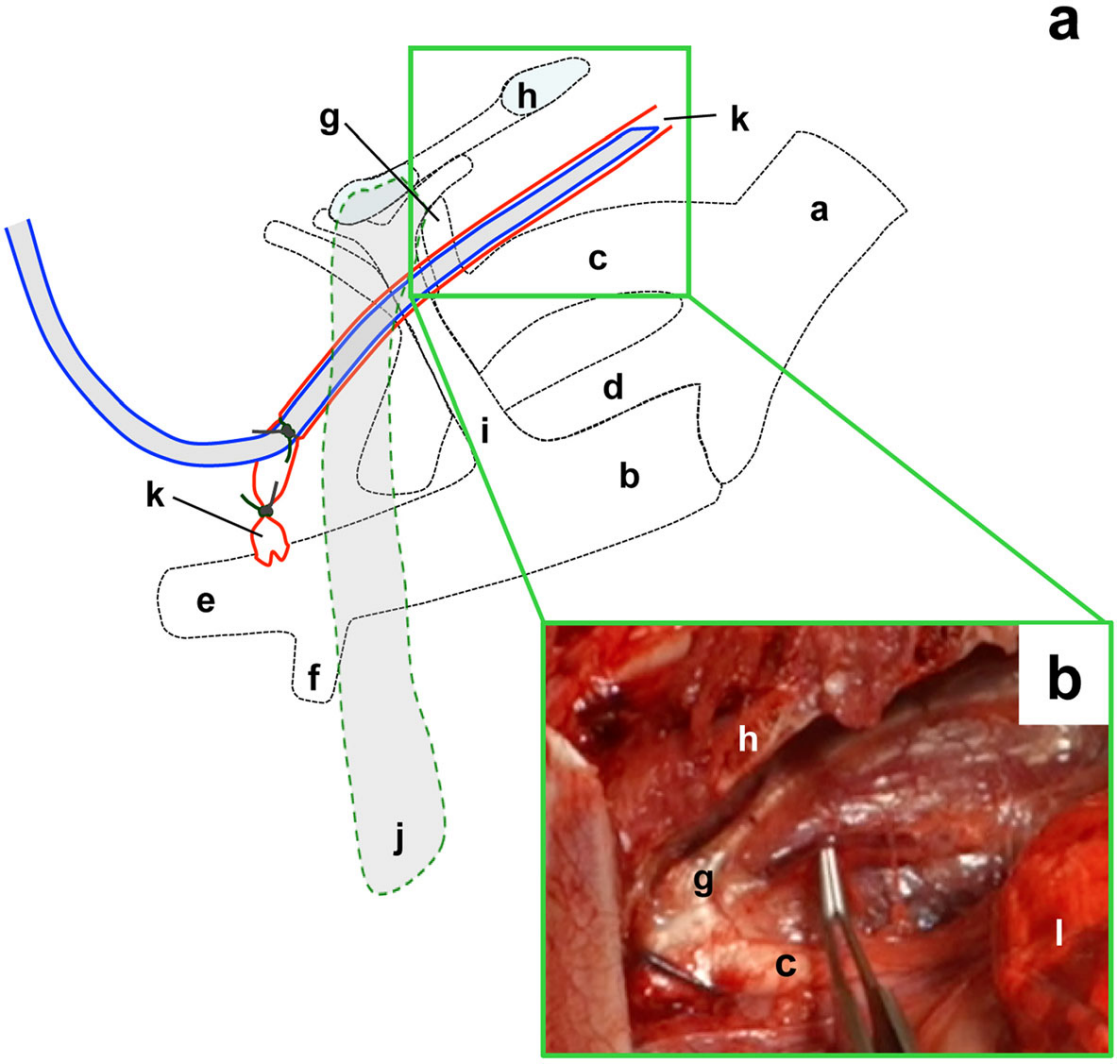
Sketch of thoracic duct cannulation (**a**) and a photograph (**b**) of a cannula in the thoracic duct at the cranial thorax on the left side. The cannula was inserted into the thoracic duct from a cut into the upstream end of the ampulla. After inserting the cannula into the thoracic duct, the cannula was then gently pushed forward until its bevelled leading end passed the junction of the costocervical trunk and subclavian artery. The bevelled end of the cannula is placed in the segment of the thoracic duct medial to the second to third rib as this position has more space and is free of major blood vessels. (**b**) Dissections were performed to confirm the insertion of a cannula in the thoracic duct following a thoracic duct cannulation surgery. This photograph of the cranial thorax shows a cannula in a segment of the thoracic duct elevated by a pair of forceps. Since the cannula was in the thoracic duct, the pressure of lifting it up did not cause the lymphatic to collapse where it was supported by the forceps. The cannula could be pushed gently far enough so that its bevelled leading end reached the segment of the thoracic duct medial to the 2^nd^ to 3^rd^ ribs. (a) thoracic aorta; (b) cranial vena cava; (c) subclavian artery; (d) brachiocephalic trunk; (e) external jugular vein; (f) subclavian vein; (g) costocervical trunk; (h) 2^nd^ rib; (i) costocervical vein; (j) first rib; (k) thoracic duct; (l) lung


The cannula was inserted into the thoracic duct from a cut made in the upstream end of the ampulla (Fig. 3). After inserting the cannula into the thoracic duct, the cannula was then gently pushed forward until its bevelled end passed the junction of the costocervical trunk and subclavian artery. The bevelled end (lead) of the cannula is best placed in a position with more space and free of major blood vessels in the cranial thorax. The segment of the thoracic duct medial to the second and the third ribs was a suitable position for the lead of the cannula. An autopsy was performed to confirm the insertion of a cannula into the thoracic duct as well as the location of the lead of a cannula in the thoracic duct immediately after the cannulation surgery. Figure 3b shows a cannula within the thoracic duct in the cranial thorax that has been elevated by a pair of forceps. Since the cannula was within the thoracic duct, the pressure of lifting the duct up did not result in a collapsed lymphatic at the contact point of the forceps Additional file 1: Video S1 and Additional file 2: Video S2.

**Additional file 1**: (The video, “lymph flowing after cannula insertion into the thoracic duct 240615.mov” demonstrated that lymph flows through the cannula following thoracic duct cannulation. The video, “cannula within the thoracic duct copy.mov” confirmed that the cannula is within the thoracic duct at the cranial thorax following thoracic duct catheterization.)


**Additional file 2**: (A video, “cannula within the thoracic duct copy.mov”, confirmed that the cannula is within the thoracic duct at the cranial thorax following thoracic duct catheterization.)

After cannula insertion into the lymphatic, a small coil of the cannula was left subcutaneously. The cannula was secured using a purse-string suture at its skin opening dorsocranial to the cranial angle of the scapula [23]. For the first surgery in a 5-week-old pig, the end of the cannula external to the animal was protected in a SILASTIC® Foley catheter (16G) that was sutured to the skin at the cannula’s exit to the skin and attached to a step-in harness (PETstock, Victoria, Australia). The use of a Foley catheter provided additional protection to the cannula and possibly the protection resulted in a longer period of lymph collection during the post-operative period. However, active movements of the pig induced more tissue damage at the skin opening since the calibre of the Foley catheter was much bigger than that of the SV28 and SV45 cannulas. In the 8-week old pig, we did not suture the Foley catheter to the cannula’s skin opening. Instead, the Foley catheter was sutured to the skin near the cannula’s skin opening. Only minor tissue responses were found at the cannula’s skin opening in this pig. The step-in harness together with the Foley catheter was secured on the animal with tubular elastic net bandages (size 3, Surgifix®). The tube for lymph collection was secured in a plastic bottle that was tightly tethered to the harness on the left side of the pig. An opening on the side of the bottle was made to allow convenient changing of the collecting tube.

**Fig. 4:**
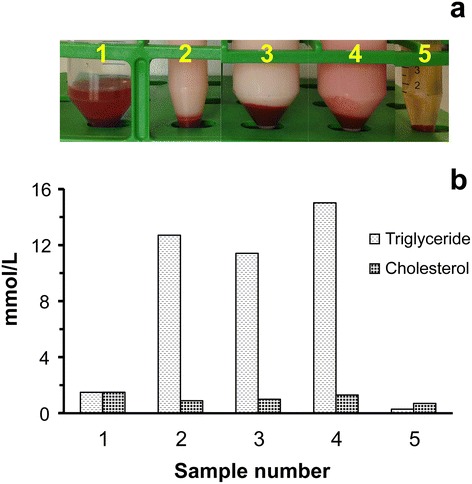
The appearance of thoracic duct lymph and interstitial fluid samples (**a**) and their corresponding levels of triglyceride and cholesterol (**b**). A cannula with an internal diameter of 0.58 mm and an external diameter of 0.96 mm (code: SV45) was inserted into the thoracic duct of an 8-week old pig. Lymph and interstitial fluid samples collected from this animal at different stages post-surgery were collected (**a**) and the levels of triglyceride and cholesterol in these samples are shown in (**b**). The sample labels are: 1: lymph from the thoracic duct, collected after about 30 h of fasting; 2–4: lymph from the thoracic duct after meals; 2: lymph harvested from 11 to 13 h post-surgery (14 mL of lymph in 2 h); 3: lymph harvested from 16 to 22 h post-surgery (42 mL of lymph in 5.5 h); 4: 23–33 h post-surgery; 5: interstitial fluid, cannula came out of the thoracic duct

The success of the thoracic duct cannulation surgery was confirmed by observing the colour of fluid collected. As presented in Fig. 4a, the supernatant of lymph samples (samples 2–4) collected from the thoracic duct at different time points after meals post-surgery were all milky. The colour of the “milky” or turbid lymph resembled that of the body fat of the animal. We also collected lymph (sample 1) from the animals during the period after successful cannulation and before the animal woke up. It was found that the supernatant of this lymph sample did not show the milky appearance of the later samples. The level of triglyceride was obviously lower in this sample (Fig. 4b) compared to the milky samples. However, no apparent differences of cholesterol levels were found between the milky and non-milky samples. Compatible results were found in another cannulated pig showing that the levels of triglyceride rather than cholesterol corresponded to the milky appearance of the lymph samples (Fig. 4). Before surgery, the animals did not have food intake for about 30 h. It was likely due to this reason that the lymph supernatant in sample 1 was not milky. When the cannula came out of the thoracic duct at day three post-surgery in this pig, the fluid collected from the cannula was not milky (sample 5). The colour of the supernatant of this sample (sample 5) looked like that of porcine sera.

**Fig. 5:**
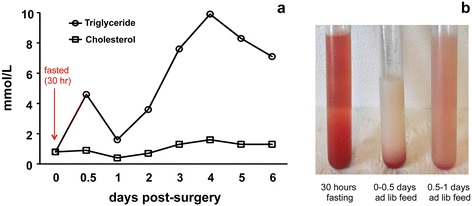
The level of triglyceride and cholesterol in the lymph collected from the thoracic duct following cannulation surgery. Triglyceride and cholesterol levels (**a**) and appearance (**b**) of lymph samples harvested from the thoracic duct of a 5-week old pig at different time points post-surgery

The levels of triglyceride and cholesterol in the lymph from the thoracic duct of a 5-week old pig were assayed for six days (Fig. 5). It was found that the amount of cholesterol was at a constant level between 0.4 and 1.6 mmol/mL. The level of triglyceride varied depending on the time for sampling in each day following unknown variations in food consumption. With regular food intake, the amount of triglyceride was between 8 and 10 mmol/mL (days 3–6). The amount of triglyceride peaked after meals and dropped dramatically without recent food intake.

It was found that lymph flow went smoothly when a cannula with a bigger calibre was placed in the thoracic duct. The rate of lymph flow was about 7 mL per hour with an SV45 cannula (internal diameter 0.58 mm; external diameter 0.96 mm) in an 8-week old pig, which weighed 17.1 kg at day one post-surgery. We also successfully inserted an SV45 cannula into the thoracic duct of a 7-week old pig, which weighed 14.7 Kg (Fig. 3b, pig euthanized after cannula insertion). Considering the smaller body size of the animal, we chose to use a smaller SV28 cannula (internal diameter 0.40 mm; external diameter 0.80 mm) in a 5-week-old pig, which weighed 9.3 kg (Fig. 5). The thoracic duct of the smaller pigs was generally smaller. It would require less skill to insert a smaller calibre cannula for animals of small sizes. However, cannula blocking by clot formation occurred frequently (once daily, always at night) in the 5-week-old pig with the smaller SV28 cannula. With intermittent cannula clotting and blocking, the total lymph volume harvested from a 5-week-old pig using an SV28 cannula from day 2 to day 5 post-surgery was 18.7, 46.5, 23.5, 16.6 and 18.0 mL respectively.

From previous experience severe cannula blocking occurred only occasionally where the SV45 cannulas were regularly applied to lymphatic cannulation surgeries in sheep, which weighed over 30 kg. We recognise that the SV45 cannulas are a more suitable choice than the SV28 cannula for the thoracic duct cannulation surgery in piglets even though the piglets only weighed around 10 Kg.

## Discussion

This is a pilot study to develop the surgical procedures for cannulating the thoracic duct at its point of entry into the external jugular vein cranial to the first rib on the left in pigs. The results in this study demonstrated that it was possible to catheterize the thoracic duct without thoracotomy and without damaging the major blood vessels, nerves and muscle bellies in 5 to 8-week old pigs. This model offers a strategy for accessing pulmonary lymph in real time and will provide a chance for evaluating in vivo pulmonary host-pathogen interplay with further modifications.

Though this surgery was successfully developed, there is still room to improve this model. In particular, to design and test a harness that can protect the external segment of the cannula from being destroyed by the animals would further ensure more robust application of the techniques described here. That is also what we will try to achieve in the subsequent studies. Pigs are strong and intransigent animals and thus additional procedures are required for protecting the cannula from any damage created by the pigs’ behaviours. Approaches such as having an external canvas pouch (“12 × 17 cm”) sutured to the skin [24] or simply by putting a solid vest on the pig are all good strategies for cannula protection. The outcome of catheter protection by applying an external custom-made canvas pouch to keep the catheters for medicine infusion in the study [24] by Swindle et al. was promising. However, many sutures were required to secure the canvas pouch to the animal. We tried a Foley catheter, which is soft and stretchy, tethered to a commercial harness for cannula protection. Compared to the application of a canvas pouch, our approach only required 2–3 sutures. Both methods were useful since they achieved the purpose of cannula protection. Our approach of using a Foley catheter for cannula protection did achieve its purpose, although the method of securing the Foley catheter to the skin at the cannula’s exit requires improvement. The success of cannula protection for long-term lymph collection using a Foley catheter should be confirmed in more animals.

This porcine thoracic duct cannulation model was primarily developed for use in pulmonary studies. However, the application of this model is not limited to pulmonary studies alone. The model could also be useful for studying diseases in which multiple organs are infected by the pathogen under study. For example, the reproductive and the respiratory tracts are the two major target organs for the porcine reproductive and respiratory syndrome virus. This model would also be a considerable boost for studying a few human diseases such as *Staphylococcus aureus* and rotavirus infections in which pigs have already been used as an animal model [4]. Catheterisation of the thoracic duct in pigs allowing for continuous in vivo, and real-time lymph sampling will also be useful in studies involving gastro-enterology and pharmacokinetics. For example, to collect lymph samples draining the cardiac region of the stomach, especially the esophagogastric junction caudal to the diaphragm, our thoracic duct cannulation model is so far the only available choice without thoracotomy. It was found that myeloid cells and T cells infiltrate into the cardiac region of porcine stomachs in *Helicobacter pylori* infection [25].

To expand the application of this model in biomedical research, we can combine this model with other methods. There were plenty red blood cells in the thoracic duct of pigs [26]. In order to avoid removing too many RBCs from the animal by continuous sampling, a lymphatico-venous shunt can be performed to drain lymph back to the circulation, similar to those described previously [11, 27]. For some studies such as those on the pharmacokinetics of medicines, molecules i.e. exosomes [28, 29] and cytokines in the body fluids in which supernatants are the target samples, we can collect lymph using a three-way valve (stopcock) that links the catheters. By draining lymph back to the circulation and using three-way stopcocks to control lymph flow, it is possible to collect lymph samples at specific arranged time points. Using this strategy, lymph samples draining the tissues may be collected at multiple time points post-treatment from the same animal instead of euthanizing a sequence of animals to obtain the tissues at each single time point.

Considering the low numbers of white cells in the peripheral afferent and efferent lymph [10], the approach we developed will be more helpful in studying the changes of components in the supernatants of efferent lymph before and after treatments. The contaminations of lipids in the lymph samples should not hinder the detection of components such as ovalbumin in the lymph supernatants when suitable methods are applied to assaying the lymph samples. Ovalbumin that was delivered to the lungs of sheep could be detected in the lymph collected from the thoracic duct using protein-specific ELISA [16]. Other components such as nitric oxide (NO), RNA and DNA can be purified using the commercially available products, such as reversed phase C18 columns [30], SV Total RNA Isolation System (Promega) and TRIzol LS Reagent (Invitrogen) depending on the aims of the research. Methods such as PCR and next-generation sequencing technologies allowing single cell sequencing are useful and sensitive tools for analyzing samples with low quantity. These technologies now make it possible to detect more information from the limited numbers of efferent lymph cells, which responded to antigen stimulation in a way that is qualitatively similar to that in sheep but smaller in magnitude [10].

## Conclusions

To obtain the kinetics of the components in the lymph would boost our understanding of the immunobiology of the draining tissues or pathobiology of the draining tissues in infections. Here, we provide a new approach to monitor the lymphatic kinetics of the thoracic duct in real-time in piglets. With further modifications of our method to achieve long-term lymph collections, we believe that this method will be a useful approach in investigating the processes of diseases such as that induced by PRRSV or *Mycoplasma hyopneumoniae* in pigs.

## Abbreviations

PRRSV: Porcine reproductive and respiratory syndrome virus
RBCs: Red blood cells
